# Healthcare Resilience in Saudi Arabia: The Interplay of Occupational Safety, Staff Engagement, and Resilience

**DOI:** 10.3390/ijerph21111428

**Published:** 2024-10-28

**Authors:** Amal Abdulmajeed Qassim, Selma Sidahmed Abedelrahim

**Affiliations:** Department of Management, Faculty of Business Administration, The University of Tabuk, Tabuk 71421, Saudi Arabia; ss.mohammed@ut.edu.sa

**Keywords:** occupational health and safety, organizational resilience, staff resilience, staff engagement

## Abstract

This research investigates the relationships between occupational health and safety (OHS), staff resilience (SR), staff engagement (SE), and organizational resilience (HOR) within Saudi Arabian hospitals. Employing a quantitative, cross-sectional design, data were collected from 127 administrative staff members working in both public and private hospitals in Saudi Arabia. Using SmartPLS to test the hypothesized relationships and mediation effects, the findings reveal that OHS significantly impacts both SR and SE, and SR significantly influences HOR. Additionally, SE significantly affects HOR. This study also confirms a partial mediation effect of SE and SR in the relationship between OHS and HOR. The model demonstrates substantial explanatory power for HOR, SE, and SR. These results underscore the critical role of OHS in fostering a resilient healthcare environment by enhancing staff engagement and resilience. This study’s implications highlight the importance of targeted interventions to improve OHS practices, promoting overall hospital resilience in alignment with Saudi Arabia’s Vision 2030.

## 1. Introduction

In today’s dynamic and sometimes fragile business climate, organizational resilience has become a key ingredient for stability, as well as for higher performance and future growth. Competitiveness and organizational agility are now considered to depend on organizational resilience, which in this study is described as an organization’s capacity to engage in early recognition, the assessment of hazards, preparation, response, and recovery operations in anticipation of gradual or abrupt changes to enable it to survive and thrive [1,2]. This concept is particularly relevant in acute care environments, where the ability to continue to function effectively when under pressure can be a matter of life and death.

Workforce health and safety are some of the crucial building blocks of organizational resilience since it is the people in organizations that are involved in business operations. Occupational health and safety is a critical concern because several occupational diseases and fatalities are witnessed in current working environments [3]. According to Aluko et al. (2016) [4], OHS involves controlling hazards in the workplace to sustain an acceptable level of risk exposure. OHS management aims to safeguard persons involved within an organization, its processes, and from adverse impacts from the organization’s products, services, and processes [3]. This protection in turn impacts on employee resilience based on the fact that employees are subjected to different kinds of and levels of occupational risks [3]. Based on Griffith and West (2013) [5], the worker is more than machinery; the worker is the life of the company because of what attitudes and actions they bring as a multiplier of openness or resistance to change. Similarly, Rook et al. (2018) [6] point out that, in the current unforgiving environment, vulnerability is vital for appropriate employees. Hence, when an organization does not incorporate the occupational health and safety practices in a form that responds to the needs of the employees, it may be compromising the employee and organizational achievements.

Previous research points to the importance of employee resilience and their interest in the development of organizational resilience. Markos et al. (2010) [7] highlight that employee engagement positively impacts efficiency, productivity, and organizational performance. Similarly, engagement—characterized by vigor, dedication, and absorption in work—has been connected to higher organizational resilience [8,9]. Kuntz et al. (2016) [10] also reveal that resilient employees help foster organizational resilience given that they are able to cope with stress and change. However, the effect of occupational health and safety (OHS) on these mediators and consequently on organizational resilience has received limited attention.

There are a few studies that concern the associations between OHS, staff engagement, staff resilience, and organizational resilience within inpatient facilities, although the research has begun exploring the benefits of resilience to the employees including variations in working days with absent healthcare staff and selected healthcare providers’ job satisfaction [11]. Furthermore, there are gaps in knowledge about individual and organizational resilience [12].

The links between OHS, staff engagement (SE), staff resilience (SR), and hospital organizational resilience (HOR) would be explored in the Saudi Arabian context in order to fill the gap. This research can be recommended to have significant implications on the Saudi healthcare system because this study aims to present policymakers and healthcare managers with practical guidelines and recommendations. These realizations might foster increased patient safety and the sustenance of a stronger and efficient health facility, which will align the country with its Vision 2030.

This study is structured as follows: the following part contains an explanation of the research strategy and methods, as well as a review of the relevant literature. The findings and analysis of the data are then presented next. The findings are then explored with an emphasis placed on the implications that are beneficial for future hospital management and regulation. In this paper’s conclusion, the limitations of this study are established, and the need to conduct further studies as well as practical implications are highlighted.

## 2. Literature Review

This literature review explores the intricate relationships between occupational health and safety (OHS), staff resilience (SR), staff engagement (SE), and hospital organizational resilience (HOR) within the context of hospitals in Saudi Arabia.

### 2.1. Theoretical Underpinnings

The theoretical foundation of this research is anchored in resilience theory and the job demands–resources (JD–R) model. This theoretical integration underscores the multifaceted impacts of OHS on both individual and organizational levels, providing a comprehensive lens through which to explore the dynamics within Saudi Arabian hospitals.

#### 2.1.1. Resilience Theory

Resilience theory emphasizes the ability of individuals and organizations to withstand, adapt to, and recover from adverse situations. Carlson et al. (2012) [13] describe resilience as a multifaceted concept encompassing robustness, resourcefulness, and adaptability, essential for maintaining functionality during crises. Kantur and İşeri-Say (2012) [2] further elaborate on this by proposing a conceptual, integrative organizational resilience framework, highlighting the interplay between cognitive, behavioral, and contextual factors. This framework underscores the importance of proactive and reactive capabilities in fostering resilience. Kantabutra and Ketprapakorn (2021) [14] build on this by discussing the dynamic and iterative nature of resilience, where organizations continuously evolve and adapt their structures and processes in response to internal and external challenges. Collectively, these perspectives illustrate that resilience is not merely a reactive mechanism but a proactive strategy that involves continuous learning, adaptation, and improvement. Resilience theory provides a comprehensive understanding of how organizations can sustain performance and thrive despite significant disruptions and uncertainties.

#### 2.1.2. The Job Demands–Resources (JD–R) Model

The job demands–resources (JD–R) model, developed by Demerouti et al. (2001) [15], is a comprehensive framework that examines how job demands and job resources interact to influence employee well-being and organizational outcomes. According to this model, job demands refer to a job’s physical, psychological, social, or organizational aspects that require sustained physical or mental effort and are therefore associated with specific physiological and psychological costs. Conversely, job resources are those physical, psychological, social, or organizational aspects of the job that help achieve work goals, reduce job demands, and stimulate personal growth and development. The model suggests that high job demands can lead to burnout and other adverse outcomes, especially when job resources are insufficient. Conversely, adequate job resources can buffer the negative impact of job demands and enhance motivation and engagement [16]. Taris and Schaufeli (2015) [17] expand on this by highlighting how job resources mitigate the adverse effects of job demands and play a crucial role in fostering employee engagement, which is critical for organizational success. Lesener et al. (2019) [18] provide a meta-analytic review of longitudinal studies supporting the JD–R model, demonstrating that job resources are significantly associated with positive work outcomes over time, while job demands are linked to burnout and other adverse outcomes. Overall, the JD–R model offers a robust framework for understanding the dynamic interplay between job demands and resources, emphasizing the importance of maintaining a balance to promote employee well-being and organizational resilience.

### 2.2. Relationship Between Occupational Health and Safety (OHS) and Staff Resilience (SR)

Occupational health and safety (OHS) refers to practices, policies, and regulations designed to prevent workplace injuries, illnesses, and accidents. This includes risk assessments, safety training, ergonomic interventions, and mental health support [19,20]. Staff resilience (SR) is the capacity of individuals to recover from adverse events, cope with stress, and adapt positively to changes and challenges in the workplace [1,10]. Resilient staff can effectively manage work-related pressures, maintain performance, and contribute positively to their work environment [21]. This resilience is influenced by cultural and national values, which shape how employees perceive and respond to organizational challenges, particularly in times of adversity or crisis [22]. Additionally, authentic leadership is critical in fostering employee resilience by providing support and promoting a positive organizational climate, which helps employees navigate workplace challenges more effectively [23]. In hospitals, resilient staff can effectively manage high workloads, emotional stress, and unexpected situations [8].

The relationship between OHS and SR involves physical safety, mental health, and organizational support. Comprehensive OHS programs protect employees from physical hazards and provide psychological support, contributing to overall well-being and resilience [12,24]. García et al. (2007) [25] find that addressing psychosocial risk factors through OHS interventions significantly improves employees’ mental health and resilience. Similarly, Gopang et al. (2017) [26] demonstrate that small- and medium-sized enterprises (SMEs) with robust OHS practices have employees who exhibit higher resilience and better performance. Barasa et al. (2018) [1] emphasize the role of organizational resilience, which can be fostered through robust OHS practices. Their systematic review highlights that organizations with strong safety cultures and proactive health measures tend to have more resilient staff, as these measures provide a stable and supportive environment. Moreover, Itzhaki et al. (2015) [21] find that exposure to workplace violence and stress in mental health nurses was mitigated by effective OHS interventions, which enhanced their resilience and life satisfaction. This study underscores the importance of OHS in managing stressors and promoting psychological resilience in high-risk environments. Based on the literature review, the following hypothesis is proposed:

**H1:**  *Higher occupational health and safety levels in hospitals (OHS) are positively associated with greater staff resilience (SR).*

### 2.3. Relationship Between Staff Resilience (SR) and Hospital Organizational Resilience (HOR)

Hospital organizational resilience refers to a hospital’s ability to anticipate, prepare for, respond to, and adapt to incremental changes and sudden disruptions to survive and thrive [2]. This concept involves robust systems, adaptive capacity, and strategic foresight to withstand crises while providing essential healthcare services [27]. Resilience is crucial for maintaining operational continuity and quality care during natural disasters, pandemics, or other significant disruptions [1,24]. The importance of organizational resilience is further highlighted by research demonstrating its positive impact on performance during the COVID-19 pandemic, showing that resilient organizations, including hospitals, can better manage and recover from significant challenges [28,29].

The relationship between staff resilience and hospital organizational resilience is integral, particularly in the high-stakes environment of healthcare. Barasa et al. (2018) [1] underscore that organizational resilience is rooted in the resilience of its members. When staff are resilient, they contribute to a supportive and dynamic organizational culture that enhances overall resilience. Staff resilience equips employees with the skills to handle stress, adapt to changes, and recover from challenges, fostering a resilient organizational framework. Itzhaki et al. (2015) [21] highlight that resilient staff are better prepared to manage job stress and maintain high levels of job satisfaction and mental health, even when exposed to workplace violence or other stressors.

This personal resilience directly influences their ability to contribute positively to the organization’s resilience by ensuring continuous and effective service delivery during crises. In healthcare settings, where unexpected events and high-pressure situations are common, resilient staff are crucial for maintaining operational stability and quality of care [8]. According to Cooke et al. (2019) [30], high-performance work systems that enhance employee resilience also contribute to increased organizational resilience. In hospitals, such systems include training programs, supportive leadership, and employee engagement initiatives. These elements enhance individual resilience and create a robust organizational culture that can withstand and adapt to crises [31]. Liang and Cao (2021) [32] demonstrate that resilient employees contribute to organizational resilience through effective coping mechanisms and managerial resilience. This interplay is crucial in hospitals where the well-being of staff directly impacts patient care and organizational effectiveness. Resilient staff are more likely to engage in proactive behaviors, collaborate effectively with colleagues, and support organizational initiatives to enhance resilience [25]. Given the relationships and empirical evidence, we hypothesize the following:

**H2:**  *Increased staff resilience (SR) is positively associated with enhanced hospital organizational resilience (HO* *R).*

### 2.4. Relationship Between Occupational Health and Safety (OHS) and Hospital Organizational Resilience (HOR)

The relationship between occupational health and safety and hospital organizational resilience is increasingly recognized as critical in ensuring the sustainability and effectiveness of healthcare services, particularly in times of crisis. Occupational health and safety in hospitals refer to the policies, procedures, and practices implemented to protect healthcare workers’ physical and mental well-being. This includes addressing hazards, providing training, ensuring adequate staffing, and promoting a workplace health and safety culture [19]. On the other hand, hospital organizational resilience pertains to the hospital’s ability to anticipate, respond to, and recover from adverse events, ensuring continuous service delivery and maintaining its core functions under stress [1]. Allende et al. (2017) [20] suggest that effective OHS practices are fundamental in mitigating risks and fostering a resilient organizational structure. They argue that hospitals with robust OHS frameworks are better positioned to withstand and recover from disruptions, enhancing their overall resilience. Additionally, the systematic review by Barasa et al. (2018) [1] highlights the multifaceted nature of resilience, identifying various strategies and interventions that strengthen organizational capacity, such as leadership support, employee training, and comprehensive safety programs. This aligns with the findings of Gopang et al. (2017) [26], who demonstrate through empirical research that effective OHS measures directly correlate with improved performance metrics and resilience in SMEs, extending the applicability of these principles to healthcare settings. This leads to the formulation of the following hypothesis:

**H3:**  *Occupational health and safety in hospitals (OHS) has a direct positive impact on hospital organizational resilience (HOR).*

### 2.5. Relationship Between Occupational Health and Safety (OHS) and Staff Engagement (SE)

Staff engagement refers to employees’ emotional commitment and involvement with their organization and its goals. Engaged employees display enthusiasm, dedication, and a readiness to surpass their basic job requirements [33,34]. This engagement is influenced by factors such as transformational leadership, job resources, and opportunities for recovery, which collectively enhance employees’ willingness to invest effort and energy in their roles [33]. A safe working environment significantly influences staff engagement. When healthcare workers feel safe and their well-being is prioritized, they are more likely to be motivated and committed to their work. This sense of security enhances their ability to engage fully in their roles, leading to overall job satisfaction [30]. Furthermore, job demands and resources are critical in determining engagement levels. High job resources, such as support and feedback, can buffer the adverse effects of job demands, reducing burnout and promoting engagement [35]. Healthcare organizations can foster a highly engaged and resilient workforce by ensuring a supportive and safe work environment. For instance, Gopang et al. (2017) [26] emphasize that effective OHS measures directly contribute to employees’ improved performance and well-being, which fosters higher engagement levels. In contrast, poor occupational health and safety can increase stress, burnout, and job dissatisfaction among hospital staff. This affects their mental and physical health and diminishes their capacity to engage positively with their work [21]. García et al. (2007) [25] find that adverse psychosocial factors and poor occupational health conditions are associated with low levels of staff engagement and increased absenteeism. Based on the reviewed literature, the following hypothesis is proposed:

**H4:**  *Occupational health and safety in hospitals (OHS) has a direct positive impact on staff engagement (SE).*

### 2.6. Relationship Between Staff Engagement (SE) and Hospital Organizational Resilience (HOR)

Studies have shown that high levels of staff engagement contribute positively to organizational resilience. Allende et al. (2017) [20] emphasize that aligning organizational pathologies with resilience indicators is crucial for sustaining organizational health and productivity. Engaged employees are more likely to exhibit proactive behaviors, adapt to changes, and support their organization through challenges. Similarly, Barasa et al. (2018) [1] highlight that fostering resilience through engaged staff can improve responses to external pressures and shocks. Brown et al. (2017) [24] demonstrate that engaged employees are vital for maintaining operational resilience during disruptions. Their findings suggest that engaged staff are better equipped to handle stress, recover quickly from setbacks, and contribute to organizational continuity. Dwomoh et al. (2020) [36] explore human resource strategies during the COVID-19 pandemic and discover that engaging staff through supportive policies and practices helps maintain business sustainability. This reinforces the idea that employee engagement is a foundational element of organizational resilience. Given the existing literature, the following is hypothesized:

**H5:**  *There is a positive relationship between staff engagement (SE) and hospital organizational resilience (HOR).*

### 2.7. Mediator Roles

Staff resilience, the ability of employees to adapt and thrive in the face of adversity, is crucial in mediating the effects of OHS on organizational resilience. Itzhaki et al. (2015) [21] explore the relationship between exposure to violence, job stress, and staff resilience among mental health nurses, highlighting how resilient staff can better cope with occupational hazards and maintain high job satisfaction and performance levels. This resilience, in turn, contributes to overall organizational resilience. García et al. (2007) [25] link adverse psychosocial factors to poor occupational health, demonstrating that addressing these factors through effective OHS measures can enhance staff resilience. Liang and Cao (2021) [32] explore how employee resilience contributes to organizational resilience through coping mechanisms and managerial resilience, suggesting a cascading effect where resilient employees bolster the organization’s overall resilience. Itzhaki et al. (2015) [21] and Kuntz et al. (2016) [10] underscore the importance of psychological resilience among healthcare workers. They suggest that occupational stressors and exposure to violence significantly impact mental health, which, in turn, affects the overall resilience of hospital systems. Creating a supportive work environment through OHS interventions reduces stress and burnout and enhances staff resilience, enabling them to better cope with challenges. This relationship is further supported by the work of Liang and Cao (2021) [32], who illustrate the role of coping mechanisms and managerial resilience in linking employee resilience to organizational resilience, thereby highlighting the interconnectedness of individual and organizational levels of resilience. Given the relationships and empirical evidence, we hypothesize the following:

**H6:**  *Staff resilience (SR) mediates the relationship between occupational health and safety (OHS) and hospital organizational resilience (HOR).*

Research indicates that staff engagement can mediate the relationship between OHS and organizational resilience. For instance, effective OHS measures can lead to higher employee satisfaction and lower stress levels, enhancing engagement [3,25]. Engaged employees are more resilient and better able to adapt to changes and challenges, contributing to overall organizational resilience [10,31]. Based on the literature, the following hypothesis is proposed:

**H7:**  *Staff engagement (SE) mediates the relationship between occupational health and safety (OHS) and organizational resilience in hospitals (OR).*

Staff engagement and resilience can mediate the relationship between OH and OR. Meintjes and Hofmeyr (2018) [37] find that perceived organizational support enhances employee engagement and resilience, which are crucial for organizational resilience. Similarly, Tonkin et al. (2018) [38] highlight the importance of employee well-being in building organizational resilience through enhanced engagement and resilience. Given these findings, we hypothesize the following:

**H8:**  *Staff engagement (SE) and staff resilience (SR) serially mediate the relationship between occupational health (OHS) and organizational resilience (OR) in hospitals in Saudi Arabia.*

## 3. Method

### 3.1. Research Design, Sample, and Population

**Figure 1:**
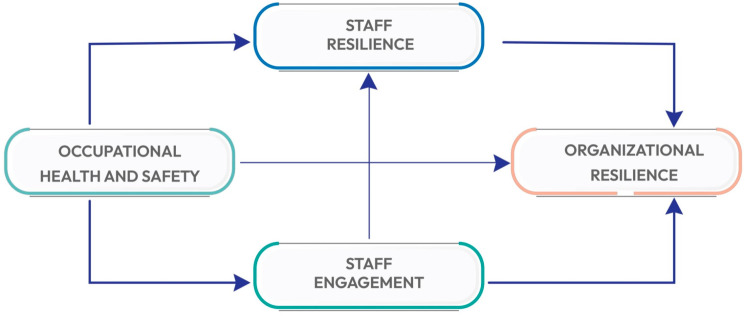
Research conceptual model.

This research investigates the relationships among occupational health and safety (OHS), staff resilience (SR), staff engagement (SE), and hospital organizational resilience (HOR) within hospitals in Saudi Arabia see Figure 1. A robust quantitative research design was utilized, adopting a quantitative approach with PLS-SEM, a sophisticated statistical tool for analyzing complex cause-and-effect relationship models that involve multiple predictors and outcomes. Data analysis was conducted using Smart PLS version 4, developed by Hair, et al., (2024) [39]. The population for this research consists of administrative staff working in public and private hospitals across Saudi Arabia. A stratified random sampling method was employed to ensure a representative sample, resulting in 127 respondents. The demographic variables describing the sample are presented in Table 1.

The demographic analysis highlights key patterns in qualifications and experience among male and female administrative staff in Saudi Arabian hospitals. Most male respondents with secondary education are aged 41–50 (26%), while graduates are mostly in the 31–40 age group (71%), and postgraduates are primarily aged 41–50 (42%). Female respondents with secondary education are mainly aged 20–30 (15%), with graduates in the 20–30 age range (79%), and postgraduates mostly aged 31–40 (32%). In terms of experience, a significant number of males have over 15 years of experience (43%), while females predominantly have less than five years (46%). Both genders show a balanced distribution in the 5–10 years’ experience category.

### 3.2. Data Collection

Data were collected using a structured questionnaire, including validated scales and measures for each variable, as shown in Table 2.

### 3.3. Data Analysis and Results

The collected data were analyzed using partial least squares (PLS) path modeling. SmartPLS version 4, developed by Ringle et al. in 2024 [39] was used to perform the data analysis, as it enables the analysis of ordinal variables [44]. In addition, it employs a component-based approach to structural equation modeling, making it highly suitable for exploratory research and applicable for confirmatory research [45]. The model has two types of variables: exogenous latent variables, which explain other constructs, and endogenous latent variables, which are the constructs being investigated [46]

### 3.4. Measurement Model

The analysis of the measurement model, as presented in Table 3, demonstrates robust reliability and validity across all constructs: occupational health and safety, organizational resilience, staff engagement, and staff resilience.

The Cronbach’s alpha values for all constructs are above the threshold of 0.7, indicating high internal consistency reliability. Specifically, occupational health and safety has a Cronbach’s alpha of 0.928, organizational resilience of 0.950, staff engagement of 0.949, and staff resilience of 0.953, signifying excellent reliability for each construct. Furthermore, the composite reliability (rho_a and rho_c) values for all constructs exceed 0.7, underscoring the constructs’ high reliability. Occupational health and safety exhibit a rho_a of 0.959 and rho_c of 0.941, organizational resilience shows a rho_a of 0.953 and rho_c of 0.959, staff engagement presents a rho_a of 0.952 and rho_c of 0.958, and staff resilience demonstrates a rho_a of 0.954 and rho_c of 0.959. These high values indicate that the measurement items consistently reflect their respective constructs.

The average variance extracted (AVE) values, which measure convergent validity, are also above the acceptable threshold of 0.5 for all constructs. Occupational health and safety has an AVE of 0.606, organizational resilience has an AVE of 0.744, staff engagement has an AVE of 0.767, and staff resilience has an AVE of 0.659. These AVE values confirm that the latent constructs capture a substantial amount of variance in the observed variables, indicating good convergent validity. According to Table 3, construct reliability and validity are demonstrated.

### 3.5. Discriminant Validity

Discriminant validity was assessed using the Fornell–Larcker criterion, which evaluates whether each construct shares more variance with its indicators than with other constructs. According to the Fornell–Larcker criterion, the square root of the AVE of each construct should be greater than the highest correlation it has with any other construct. The results are presented in Table 4 below.

The diagonal elements (bolded) represent the square root of the AVE for each construct, while the off-diagonal elements represent the correlations between constructs. The square roots of the AVEs for OHS (0.778), OR (0.863), SE (0.876), and SR (0.812) are all greater than their respective inter-construct correlations. This indicates that each construct shares more variance with its indicators than other constructs, confirming discriminant validity. These results support the distinctiveness of OHS, OR, SE, and SR within the model, thus providing evidence for the validity of the measurement model according to the Fornell–Larcker criterion.

### 3.6. Structural Model Measurement

The structural model analysis focuses on assessing the overall explanatory power, path coefficients (β), and significance levels to evaluate the relationships between the constructs. The explanatory power is measured using the R^2^ value, which indicates the proportion of variance in the dependent variable explained by the independent variables. High R^2^ values suggest a strong explanatory power of the model. Path coefficients (β) represent the strength and direction of the relationships between constructs, while significance levels determine the statistical significance of these relationships.

Collinearity statistics, represented by the variance inflation factor (VIF), are crucial for assessing multicollinearity among predictor variables. A VIF value below 5 generally indicates that multicollinearity is not a concern. In Table 5, the VIF values for the paths range from 1.000 to 2.666, all of which are well below the critical threshold of 5, indicating that multicollinearity is not an issue and that the estimates of the path coefficients are reliable.

**Figure 2:**
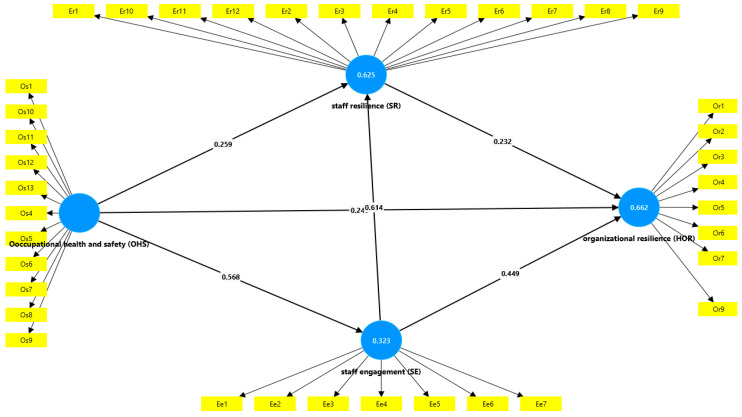
Structural model.

The values in Table 5 demonstrate that the predictor variables in the model do not exhibit problematic levels of collinearity, ensuring that the path coefficients derived from the structural model are robust and interpretable see Figure 2. The analysis confirms that occupational health and safety, along with staff engagement and resilience, significantly contribute to explaining organizational resilience, underscoring the importance of these factors in fostering a resilient healthcare environment.

### 3.7. The Model’s Explanatory Power

The model’s explanatory power is assessed using the coefficient of determination (R-squared), which indicates the proportion of variance in the dependent variables that is predictable from the independent variables. As presented in Table 6, the R-squared value for organizational resilience (OR) is 0.662, with an adjusted R-squared of 0.654, suggesting that the model can explain approximately 66.2% of the variance in organizational resilience. This high value indicates strong explanatory power for this construct. For staff engagement (SE), the R-squared value is 0.323 and the adjusted R-squared is 0.317, indicating that the model accounts for 32.3% of the variance in staff engagement, reflecting moderate explanatory power. Staff resilience (SR) shows an R-squared value of 0.625 and an adjusted R-squared of 0.619, meaning that the model explains 62.5% of the variance, which denotes substantial explanatory power. Overall, these R-squared values demonstrate that the model has good explanatory power, particularly for organizational resilience and staff resilience, highlighting the model’s robustness in explaining the variance in these key constructs within the context of occupational health and safety, staff resilience, and staff engagement. See Table 6.

### 3.8. Path Coefficients and Hypothesis Testing

This section, based on Table 7, provides an analysis of the path coefficients and hypothesis testing, which are critical in understanding the relationships between occupational health and safety (OHS), staff resilience (SR), staff engagement (SE), and hospital organizational resilience (HOR) within the studied context.

#### 3.8.1. Hypothesis Testing

Hypothesis H1, which posits that occupational health and safety (OHS) positively affects staff resilience (SR), is strongly supported by the data. The original sample (O) value is 0.608, indicating a positive relationship between OHS and SR. The sample mean (M) is 0.616, with a standard deviation (STDEV) of 0.088, which reflects the variability in this relationship in the sample. The T statistics value of 6.872 (|O/STDEV|) is significantly above the common threshold of 1.645 for significance in a one-tailed test, suggesting strong evidence against the null hypothesis. The *p*-value of 0.000 further confirms the statistical significance of this relationship. Given these results, hypothesis H1 is accepted, indicating that better occupational health and safety practices significantly enhance staff resilience. This finding underscores the importance of prioritizing OHS in fostering a resilient workforce, which is particularly crucial in the demanding environment of healthcare settings in Saudi Arabia.

The analysis validates hypothesis H2, which proposes that staff resilience (SR) positively influences organizational resilience (OR). The original sample (O) value of 0.232 indicates a positive association between SR and OR. The sample mean (M) is 0.221, with a standard deviation (STDEV) of 0.096, highlighting the variability within the data. The T statistic of 2.416 (|O/STDEV|) exceeds the threshold of 1.645 for a one-tailed test, providing robust evidence against the null hypothesis. Additionally, the *p*-value of 0.008 signifies statistical significance. Therefore, hypothesis H2 is accepted, demonstrating that enhanced staff resilience significantly contributes to greater organizational resilience. This result emphasizes the critical role of fostering staff resilience to strengthen the overall resilience of healthcare organizations, aligning with broader efforts to enhance healthcare quality and stability in Saudi Arabia.

The analysis strongly supports hypothesis H3, which posits that occupational health and safety (OHS) positively influences organizational resilience (OR). The original sample (O) value is 0.636, indicating a robust positive relationship between OHS and OR. The sample mean (M) is 0.646, with a standard deviation (STDEV) of 0.072, suggesting consistent findings. The T statistic 8.845 (|O/STDEV|) significantly exceeds the critical value 1.645 for a one-tailed test, providing substantial evidence against the null hypothesis. The *p*-value of 0.000 confirms the statistical significance of this relationship. Therefore, hypothesis H3 is accepted, demonstrating that effective occupational health and safety measures significantly enhance organizational resilience. This underscores the importance of OHS practices in building a resilient healthcare system, which is crucial for improving the quality and stability of healthcare services in Saudi Arabia. The findings align with the broader goals of national health policies, emphasizing the need for robust OHS frameworks to support organizational resilience in the healthcare sector.

Hypothesis H4, which asserts that occupational health and safety (OHS) positively impacts staff engagement (SE), is confirmed by the data analysis. The original sample (O) value of 0.568 signifies a substantial positive effect of OHS on SE. The sample mean (M) is 0.577, with a standard deviation (STDEV) of 0.087, indicating a reliable and consistent relationship. The T statistic of 6.553 (|O/STDEV|) greatly exceeds the critical value of 1.645 for a one-tailed test, providing robust evidence against the null hypothesis. The *p*-value of 0.000 further reinforces the statistical significance of this finding. Consequently, hypothesis H4 is accepted, demonstrating that effective occupational health and safety measures significantly enhance staff engagement. This result highlights the critical role of OHS in fostering a motivated and engaged workforce, which is essential for the overall performance and resilience of healthcare organizations in Saudi Arabia. The findings suggest that improving OHS practices can lead to higher levels of staff engagement, contributing to a more committed and productive workforce, thereby aligning with the objectives of national health initiatives and policies.

Hypothesis H5, which posits that staff engagement (SE) positively influences organizational resilience (OR), is supported by the data analysis. The original sample (O) value is 0.591, indicating a strong positive effect of SE on OR. The sample mean (M) is 0.580, with a standard deviation (STDEV) of 0.090, reflecting a consistent and reliable relationship between these constructs. The T statistic of 6.593 (|O/STDEV|) significantly exceeds the critical value of 1.645 for a one-tailed test, providing compelling evidence to reject the null hypothesis. The *p*-value of 0.000 further confirms the statistical significance of this result. Therefore, hypothesis H5 is accepted, demonstrating that higher levels of staff engagement contribute significantly to organizational resilience. This finding underscores the importance of fostering engagement among staff to enhance the resilience of healthcare organizations in Saudi Arabia. Engaged employees are likely to be more proactive, adaptive, and supportive. These are key attributes for building resilient healthcare institutions capable of surviving various operational challenges and improving healthcare delivery.

#### 3.8.2. Assessment of the Mediator Construct

Hypothesis H6 examines the mediating role of staff resilience (SR) in the relationship between occupational health and safety (OHS) and organizational resilience (OR). The analysis supports this hypothesis, with an original sample (O) value of 0.060, indicating a positive indirect effect of OHS on OR through SR. The sample mean (M) is 0.057, with a standard deviation (STDEV) of 0.030. The T statistic of 2.012 (|O/STDEV|) exceeds the critical value of 1.645 for a one-tailed test, providing significant evidence to reject the null hypothesis. The *p*-value of 0.022 confirms the statistical significance of this mediating effect. Therefore, hypothesis H6 is accepted, indicating that staff resilience partially mediates the relationship between occupational health and safety and organizational resilience. This finding highlights the critical role of staff resilience in translating the benefits of occupational health and safety measures into enhanced organizational resilience.

Hypothesis H7 posits that staff engagement (SE) mediates the relationship between occupational health and safety (OHS) and staff resilience (SR). The analysis confirms this hypothesis with an original sample (O) value of 0.349, indicating OHS’s strong positive indirect effect on SR through SE. The sample mean (M) is 0.343, with a standard deviation (STDEV) of 0.055. The T statistic of 6.329 (|O/STDEV|) is significantly above the critical value of 1.645 for a one-tailed test, providing substantial evidence to reject the null hypothesis. The *p*-value of 0.000 further confirms the statistical significance of this mediating effect. Consequently, hypothesis H7 is accepted, demonstrating that staff engagement significantly mediates the relationship between occupational health and safety and staff resilience. This result underscores the importance of fostering an engaging work environment to enhance staff resilience.

#### 3.8.3. Assessment of the Serial Mediators Construct

The research summarized in Table 8 assessed the serial mediation effect of staff engagement and staff resilience in the relationship between occupational health and safety (OHS) and organizational resilience (OR). The total effect of OHS on OR was significant (β = 0.636, *p* < 0.005), indicating a strong direct relationship. The direct effect of OHS on HOR remained significant (β = 0.240, *p* < 0.005), suggesting that OHS directly influences OR even in the presence of mediators. The indirect effect of OHS on OR through staff engagement and staff resilience was found to be 0.081 with a T statistic of 2.148 and a *p*-value of 0.016, indicating significance. However, this mediation effect was deemed partial, as the direct effect still held considerable influence. The confidence interval for the indirect effect ranged from 0.019 to 0.143, supporting the presence of mediation. Despite the partial mediation, these findings emphasize that while staff engagement and resilience mediate the impact of OHS on OR, there remains a significant direct effect of OHS on OR. This underscores the importance of OHS in fostering organizational resilience, both directly and indirectly, through its influence on staff engagement and resilience.

## 4. Discussion

Based on the postulated hypothesis H1, staff resilience is boosted with enhanced workplace health and safety procedures. Based on this research, OHS should be accorded the highest importance for building a strong workforce, especially when working under the challenging Saudi Arabian healthcare settings. This is with respect to resilience theory because the improved OHS procedures notably raise the levels of organizational employee resilience. Kantur and İşeri-Say (2012) [2] define resilience as the capability of individuals and organizations to bounce back when faced with adversity and survive and succeed in difficult circumstances. In the context of healthcare where demands from jobs are high, authoritative and definitive OHS measures can also have positive impacts in as much as they help motivate staff to develop the essential strength to be able to carry out their responsibilities in the healthcare team. The findings endorse the theoretical framework described by Carlson et al. (2012) [13] where resilience is the capability to sustain functionality amidst hardships with the help of protection factors such as safety and health measures. This study provides evidence for the supposition that investments in OHS are essential for developing resilience in employees, stress management, and the ability to recover from frailty, which is very important for Saudi Arabian healthcare employees.

Further, with relation to the previous literature, these results are coherent and support OHS as a core component in developing strong organizations [1,20,24]. The studies of Cooke et al. (2019) [30] and Itzhaki et al. (2015) [21] also help substantiate the idea that the emphasis placed on health as well as safety may prove beneficial in strengthening the staff’s engagement or even give effective coping strategies for challenged organizational performances. Coupled with this, other authors including Gopang et al. (2017) [25] and Sull et al. (2015) [8] opine that strong OHS practices safeguard workers and foster organizational values that enhance the ability to cope with the high levels of pressure characteristic of a healthcare setting. For this reason, this evidence indicates that healthcare organizations in Saudi Arabia could enhance the well-being and performance of staff as well as the organization by investing in OHS and, thus, lead to the better delivery of healthcare services and improved patient satisfaction.

That hypothesis H2 was accepted points to the fact that there is a much stronger relationship between organizational resilience and the resilience of the staff. This conclusion is particularly consistent with resilience theory because resilience theory asserts that it is important to strengthen staff resilience in an effort to enhance healthcare organizational resilience. This is in harmony with other measures that have been taken with a view of enhancing the stability and quality of healthcare in Saudi Arabia. These findings concur with comparable research that underscores the role of organizational and individual durability in maintaining effective functioning in healthcare settings [1,20,24]. For instance, it highlights that when employees are more resistant, there is a greater potential to overcome stress-related factors and challenges in the course of delivering healthcare services to the targeted populations, which in due course builds up and creates capacity in the healthcare organizations [2,10,47].

Resilience at the employee level is a cornerstone of organizational resilience, especially in a diverse environment such as that of the healthcare sector [14]. This is in line with Kantur and İşeri-Say’s (2012) [2] integrative framework that focuses on the application aspect of personal survival. At an individual level, this resilience ensures organizations can continue keeping operations going and deliver high performance regardless of the prevailing circumstances. Furthermore, the proposition in the literature that offering power to the employees through skill enhancement and focal leadership may tremendously enhance organizational protection also receives emphasis from the concept of creative self-efficacy that enhances resilience and performance as pointed out by Prayag and Dassanayake (2023) [48] Thus, based on the study by Prayag et al. (2020) [49] on the relationship between psychological resilience, organizational resilience, and life satisfaction, the importance of a strong workforce is stressed to maintain the performance standards and become more robust in operating under pressure.

Hypothesis H3, which claims that OHS has a positive impact on OR, could be supported by the analysis. This has supported the need to focus on OHS practices in enhancing the mission of developing a strong framework of healthcare facilities that will in turn contribute towards the enhancement in the quality and sustenance of healthcare services in the Kingdom of Saudi Arabia. This finding supports resilience theory and is in congruence with the other literature on organizational resilience and health and safety practices. For example, Allende et al. (2017) [20] note the synchronization of organizational resilience indices with the concepts of health and security as a technique to avoid organizational pathologies. Barasa et al. (2018) [1] agree with the need to foster resilience through strong healthcare policies, while Brown et al. (2017) [24] explain how measures of safety enhance the viability of critical infrastructures. Carlson et al. (2012) [13] also observe that it is possible to develop organizational resilience through factors which minimize operational risks such as health and safety. In the perspective of Saudi Arabian healthcare, this result re-establishes the fact that when OHS measures are a priority, they work as a barrier; halting all the operation threats and offering the foundation on which hospitals can establish that ensure that they will remain operational under pressures.

In addition, in their respective studies, Gopang et al. (2017) [26] and Cooke et al. (2019) [30] establish that high levels of resilience and performance are positively related to high-performance work systems including OHS. Based on these results, it is possible to state that companies may construct a more effective workforce that can counterbalance and adapt to the different sorts of problems with the help of OHS. Further, the previous research also establishes the link between OHS management and organizational resilience, which is in line with the objectives of the Saudi Arabian national health policies and guidelines on the importance of developing adequate OHS management standards for healthcare organizations. Therefore, hypothesis H4 is supported, suggesting that both comprehensive OHS practices improve staff engagement and are congruent with JD-R theory. In addition to helping relieve job demands, resources such as OHS contribute to enhancing employee outcomes by creating a supportive context that promotes well-being, according to Demerouti and Bakker (2011) [16]. It is postulated that the healthcare workers’ engagement levels rise when they conclude that their illness and safety are valued by their organization, resulting in higher performance and lower burnout levels according to Taris and Schaufeli (2015) [17].

This implementation of OHS painted a better picture for the future of healthcare management and organizational psychology. Also, Ghoudarzi et al. (2019) [50] stressed that health and safety management was related positively to job satisfaction. Gyensare et al. (2019) [51] also offer evidence from the SME sector in Ghana to indicate the need for effective occupational health and safety management to enhance high levels of employee engagement. Cooke et al. (2019) [30] also explain that high-performance work systems link with engagement and suggest that safety and health at work boost engagement. Gopang et al. (2017) [26] suggested that measures of occupational health and safety enhance performance in SMEs, which is in line with the positive effect of OHS on SE in this research. This means that the study results can extend to other contexts of the organizations. In the Saudi Arabian context of healthcare, improvement in OHS strategies can bring drastic changes in the employees’ engagement levels and overall organizational capability that helps in creating a better healthcare system based on the principles of Vision 2030.

The results of the data analysis for Hypothesis H5 which predicted that the ‘staff engagement’ (SE) variable enhances organizational resilience (OR) have been found valid in the current study. This study emphasizes the need for enhancing staff participation in the development of healthcare organizational resilience in Saudi Arabia. When employees are engaged, they are likely to be proactive, versatile, and responsive, which are essential qualities for creating sturdy healthcare organizations that can handle every aspect of their operation as well as enhance general healthcare services. This finding is supportive of the literature on involvement as well as organizational resilience. For instance, Allende et al. (2017) [20] point out that to enhance overall organizational resilience, behaviors should be aligned with resilience factors. While Brown et al. (2017) [24] focus on the importance of employee engagement in enhancing the resilience of critical infrastructure, Barasa et al. (2018) [1] discuss vital elements of building resilience within the context of the healthcare sector.

Further, conceptual work by Cooke et al. (2019) [30] and Kuntz et al. (2016) [10] offer evidence that employees’ engagement contributes to higher levels of organizational resilience, which is defined as the ability and capacity of an organization to sustain high levels of organizational performance under various forms of stress. Further, Prasongthan (2022) [52] notes that job security and employee engagement are vital practices to improve organizational resilience during or after the outbreak of a virus such as COVID-19, while Ning and Tantasanee (2023) [53] show how to develop tactical plans to promote the concept of employee engagement and organizational resilience in organizational development. Collectively, this research aligns with the JD-R concept and confirms the key connection between organizational resilience and employees’ engagement, therefore underlining the importance of deliberate efforts to strengthen such feelings about healthcare organizations’ employees. As pointed out by Lesener et al. (2019) [18], it was established that engagement could mediate the relationship between work resources and organizational gains including resilience. These attributes point to engagement as possessing the ability to enhance organizational resilience by promoting proactive, flexible, and dedicated people who are ready to cope with change. The current study supports this perspective, which shows that through improving job resources like OHS, healthcare organizations can promote staff engagement and ultimately foster organizational responsiveness to dynamic situations.

Hypothesis H6 is thus supported, showing that staff resilience partly mediates the relationship between OHS and OR. This has shown that staff resilience is a fundamental facet of applying the gains in occupational health and safety in improving organizational resilience. This raises the concern of policies and interventions to boost occupational health and safety in Saudi Arabian hospitals and concurrently foster staff well-being or enhance the capacity and flexibility of the Saudi Arabian healthcare system. This aligns with the literature on the importance of safety culture and reserve in enhancing organizational performance and readiness for emergencies [1,20,24]. In particular, the presence of OHS intervention together with the staff well-being promotion approach is critical for building organizational staff resilience within healthcare settings since both were rated as having a positive impact in the above-stated studies.

This study substantiates the mediating role of staff engagement (SE) in the OHS and staff resilience (SR) relationship. The outcome highlights the importance of creating a stimulating workplace environment to boost staff resistance. Enhancing occupational health and safety practices in Saudi Arabian hospitals can increase staff participation, making healthcare personnel more resilient when facing challenges. This outcome is consistent with the other studies that underscore the role of participation in promoting organizational resilience as shown in the papers by Barasa et al. (2018) [1] and Kuntz et al. (2016) [10]. Moreover, it builds on the research by Meintjes and Hofmeyr (2018) [37] where they emphasize on perceiving organizational support and its relation with employee engagement, stating that a safe organization fosters employee strength. Thus, it remains important for hospital management to also address safety standards as well as promotion in order to build a stronger workforce in readiness to face various challenges in the workplace as well as stress factors.

This study evaluated the serial mediation role of staff engagement and staff resilience in the relationship between occupational health and safety (OHS) and organizational resilience (OR). This partial mediation shows that while staff involvement and resilience act as major mediators of the association between OHS and OR, OHS still accounts for a huge proportion of the variance in OR. The data support both JD-R theory and resilience theory. As such, the results presented in this study corroborate both resilience theory and the JD-R theory. According to Kantur and İşeri-Say (2012) [2], resilience as an organizational variable acts as a mediator through which organizational resources like OHS positively impact organizational outcomes. Similarly, according to Demerouti et al. (2001) [15], job resources, including OHS, not only reduce burnout, but also promote the development of individual critical resources, including resilience and engagement, and thus enhance organizational resilience. The relationship between OHS and organizational resilience is moderated by staff engagement and resilience and is in line with the notion that resources such as OHS are necessary for the reduction in job demands and improvement in both engagement and resilience among employees [18]. It is also in line with the theoretical concepts outlined by Allende et al. (2017) [20] and Kantur and İşeri-Say (2012) [2] on organizational resilience indicators and frameworks. Barasa et al. (2018) [1] and Brown et al. (2017) [24] also highlight that the propensity by organizations to enhance organizational results can be anchored on resilience. In addition, studies by Cooke et al. (2019) [30], Meintjes and Hofmeyr (2018) [37], Sholikhah et al. (2021) [54], and Ojo et al. (2021) [55] confirm the connection between organizational performance, resilience, and employee engagement, especially in strengthening the concept of building healthy and sustainable healthcare systems.

The coordinated conclusions drawn from this research project demonstrate the imperative of OHS for enhancing organizational and personal workplace adaptation, as well as consequent organizational performance and viability in the healthcare facility. These broad suggestions underscore the importance of aligning OHS with tactics that foster staff well-being and overall organizational readiness for stress exposure.

## 5. Conclusions and Recommendations

This research provides significant insights into the intricate relationships between occupational health and safety (OHS), staff engagement, staff resilience, and organizational resilience (OR) within Saudi Arabian hospitals. The findings demonstrate that OHS exerts a strong direct effect on OR, underscoring the critical importance of robust safety measures in enhancing the resilience of healthcare organizations. Specifically, the results show that 67.1% of the variance in HOR can be attributed to factors related to OHS, indicating a substantial impact. Additionally, staff engagement and staff resilience serve as partial mediators in this relationship, highlighting their pivotal role in converting effective OHS practices into improved organizational outcomes. This partial mediation suggests that while OHS directly enhances organizational resilience, its impact is amplified when staff are engaged and resilient, reinforcing the need for a holistic approach to workplace safety and employee well-being.

The implications of these findings are particularly pertinent in the context of Saudi Arabia’s Vision 2030, which aims to transform the nation’s healthcare system to be more efficient, effective, and resilient. By prioritizing OHS initiatives, hospital administrators and policymakers can foster a safer and more supportive work environment, enhancing staff engagement and resilience. This approach improves organizational resilience and ensures high standards of patient care and better health outcomes. By doing so, Saudi Arabian hospitals can achieve the dual objectives of safeguarding employee well-being and improving organizational resilience, ultimately contributing to the broader goals of Vision 2030.

The healthcare sector in Saudi Arabia faces distinct challenges, including rapid population growth, a high prevalence of chronic diseases, and the continuous need to upgrade healthcare infrastructure and services. Implementing robust occupational safety measures is crucial in addressing these challenges by ensuring that healthcare workers are protected, engaged, and resilient. This, in turn, can lead to better patient outcomes and more efficient healthcare delivery.

Furthermore, fostering a culture of safety and resilience within hospitals aligns with the broader national goals of improving public health, enhancing healthcare efficiency, and maintaining high standards of care throughout the Kingdom. By prioritizing occupational safety and recognizing its positive impacts on staff engagement and resilience, Saudi hospitals can develop a more robust and adaptable healthcare system capable of meeting current and future demands. Several practical recommendations can be made to enhance hospital organizational resilience through improved occupational safety, staff engagement, and staff resilience in alignment with the goals of Vision 2030.

First, implementing comprehensive occupational safety programs tailored to the specific needs of Saudi healthcare settings is crucial. These programs should address physical, psychological, and environmental hazards to ensure a safe working environment for all healthcare workers. Regular updates and the strict enforcement of safety protocols are essential.

Second, fostering a supportive work environment is vital to enhance staff engagement. This can be achieved through regular feedback, recognition programs, and professional development opportunities. Communication between staff and management ensures that concerns and suggestions are promptly addressed.

Third, building staff resilience should be prioritized by providing training and resources that help staff develop effective coping strategies and resilience skills. Stress management workshops, resilience training programs, and access to mental health support are recommended.

Promoting work–life balance through flexible working hours and personal support initiatives is also important. Aligning occupational safety initiatives with the broader Vision 2030 objectives ensures a cohesive approach to improving healthcare quality and resilience. Involving staff in planning and implementing OHS initiatives fosters ownership and accountability. Regular assessments of OHS measures’ effectiveness and their impact on staff engagement and resilience, using data-driven adjustments, are essential for continuous improvement.

Lastly, expanding research to include a broader range of healthcare professionals and exploring the role of organizational culture and external factors will provide deeper insights. By implementing these recommendations, Saudi hospitals can build a more resilient, engaged, and safe workforce, ultimately leading to improved healthcare outcomes and a stronger healthcare system capable of meeting future challenges.

This research has several limitations. The focus on administrative staff means that future research may need to pay more attention to the perspectives of other vital healthcare professionals, such as nurses and doctors, whose resilience and engagement are also crucial for overall hospital resilience. Additionally, the relatively small sample size may limit the generalization of findings across diverse healthcare settings.

## Figures and Tables

**Table 1:** Demographics of the respondents.

			AGE			
			20–30	31–40	41–50	More than 50 Years
			%	%	%	%
Qualifications	Secondary education	Male	0%	0%	26%	14%
	Graduate		100%	71%	32%	71%
	Postgraduate		0%	29%	42%	14%
	Secondary education	Female	15%	3%	0%	0%
	Graduate		79%	65%	50%	50%
	Postgraduate		6%	32%	50%	50%
Experience	Less than five years	Male	75%	18%	11%	14%
	5–10 years		25%	24%	21%	14%
	10–15 years		0%	29%	16%	0%
	More than 15 years		0%	29%	53%	71%
	Less than five years	Female	85%	18%	20%	0%
	5–10 years		9%	47%	10%	50%
	10–15 years		0%	29%	20%	0%
	More than 15 years		6%	6%	50%	50%

**Table 2:** Study instrument structure.

Constructs	No. of Items	References
Occupational health and safety (OHS)	13	Gonçalves, L., Sala, R., & Navarro, J. B. (2022) [21]. Makori, E. M., Thuo, J. K., & Wanyama, K. W. (2012) [40].
Organizational resilience (OR)	9	Kantur, D., & Say, A. I. (2015) [41].
Staff engagement (SE)	7	Shrotryia, V. K., & Dhanda, U. (2020) [42]
Staff resilience (SR)	12	Connor, K. M., & Davidson, J. R. (2003) [43]

**Table 3:** Construct reliability and validity.

	Cronbach’s Alpha	Composite Reliability (rho_a)	Composite Reliability (rho_c)	Average Variance Extracted (AVE)
Occupational health and safety (OHS)	0.928	0.959	0.941	0.606
Organizational resilience (OR)	0.950	0.953	0.959	0.744
Staff engagement (SE)	0.949	0.952	0.958	0.767
Staff resilience (SR)	0.953	0.954	0.959	0.659

**Table 4:** Discriminant validity.

Fornell–Larcker Criterion	Occupational Health and Safety (OHS)	Organizational Resilience (OR)	Staff Engagement (SE)	Staff Resilience (SR)
Occupational health and safety (OHS)	0.778			
Organizational resilience (OR)	0.636	0.863		
Staff engagement (SE)	0.568	0.762	0.876	
Staff resilience (SR)	0.608	0.720	0.761	0.812

**Table 5:** Collinearity statistics (VIF).

	VIF
Occupational health and safety (OHS) → organizational resilience (OR)	1.655
Occupational health and safety (OHS) → staff engagement (SE)	1.000
Occupational health and safety (OHS) → staff resilience (SR)	1.476
Staff engagement (SE) → organizational resilience (OR)	2.481
Staff engagement (SE) → staff resilience (SR)	1.476
Staff resilience (SR) → organizational resilience (OR)	2.666

**Table 6:** Coefficient of determination (R-squared).

	R-Squared	R-Squared Adjusted
Organizational resilience (OR)	0.662	0.654
Staff engagement (SE)	0.323	0.317
Staff resilience (SR)	0.625	0.619

**Table 7:** Path coefficients and hypothesis testing.

	Original Sample (O)	Sample Mean (M)	Standard Deviation (STDEV)	T Statistic (|O/STDEV|)	*p*-Value	Status
H1: Occupational health and safety (OHS) → staff resilience (SR)	0.608	0.616	0.088	6.872	0.000	Accepted
H2: Staff resilience (SR) → organizational resilience (OR)	0.232	0.221	0.096	2.416	0.008	Accepted
H3: Occupational health and safety (OHS) → organizational resilience (OR)	0.636	0.646	0.072	8.845	0.000	Accepted
H4: Occupational health and safety (OHS) → staff engagement (SE)	0.568	0.577	0.087	6.553	0.000	Accepted
H5: Staff engagement (SE) → organizational resilience (OR)	0.591	0.580	0.090	6.593	0.000	Accepted
Assessment of the mediator construct						
H6: Occupational health and safety (OHS) → staff resilience (SR) → organizational resilience (OR)	0.060	0.057	0.030	2.012	0.022	Accepted
H7: Occupational health and safety (OHS) → staff engagement (SE) → staff resilience (SR)	0.349	0.343	0.055	6.329	0.000	Accepted
Assessment of the serial mediators construct						
H8: Occupational health and safety (OHS) → staff engagement (SE) → staff resilience (SR) → organizational resilience (OR)	0.081	0.077	0.038	2.148	0.016	Accepted

**Table 8:** Assessment of the serial mediators.

Total Effect Occupational Health and Safety (OHS) → Organizational Resilience (OR)	Direct Effect Occupational Health and Safety (OHS) → Organizational Resilience (OR)	Relationship	Indirect Effect	Confidence Interval		T Statistic	*p*-Value	Results
0.636; *p* < 0.005	0.240; *p* < 0.005	H8: Occupational health and safety (OHS) → staff engagement (SE) → staff resilience (SR) → organizational resilience (OR)	0.081	Lower Bound	Upper Bound	2.148	0.016	Partial mediation

## Data Availability

The results of this study are derived from surveys conducted as part of this research. Due to privacy and ethical considerations, the raw data, which contain potentially identifiable information, have not been made publicly available. However, the authors have prepared aggregated data that exclude any personal or sensitive details and can be shared. Researchers interested in accessing these data can contact the corresponding author with a reasonable request, subject to ethical standards and relevant privacy regulations. The survey instrument and methodology used in this study were carefully designed to ensure comprehensive and accurate insights into the research objectives.
